# The Hepatitis B Virus Core Variants that Expose Foreign C-Terminal Insertions on the Outer Surface of Virus-Like Particles

**DOI:** 10.1007/s12033-015-9895-9

**Published:** 2015-10-07

**Authors:** Andris Dishlers, Dace Skrastina, Regina Renhofa, Ivars Petrovskis, Velta Ose, Ilva Lieknina, Juris Jansons, Paul Pumpens, Irina Sominskaya

**Affiliations:** Protein Engineering Department, Latvian Biomedical Research and Study Centre, Ratsupites Str 1, Riga, 1067 Latvia

**Keywords:** Hepatitis B virus, Core protein, C-terminal domain, preS1 sequence, Immunogenicity, Virus-like particles

## Abstract

The major immunodominant region (MIR) and N-terminus of the hepatitis B virus (HBV) core (HBc) protein were used to expose foreign insertions on the outer surface of HBc virus-like particles (VLPs). The additions to the HBc positively charged arginine-rich C-terminal (CT) domain are usually not exposed on the VLP surface. Here, we constructed a set of recombinant HBcG vectors in which CT arginine stretches were substituted by glycine residues. In contrast to natural HBc VLPs and recombinant HBc VLP variants carrying native CT domain, the HBcG VLPs demonstrated a lowered capability to pack bacterial RNA during expression in *Escherichia coli* cells. The C-terminal addition of a model foreign epitope from the HBV preS1 sequence to the HBcG vectors resulted in the exposure of the inserted epitope on the VLP surface, whereas the same preS1 sequences added to the native CT of the natural HBc protein remained buried within the HBc VLPs. Based on the immunisation of mice, the preS1 epitope added to the HBcG vectors as a part of preS1(20–47) and preS1phil sequences demonstrated remarkable immunogenicity. The same epitope added to the original C-terminus of the HBc protein did not induce a notable level of anti-preS1 antibodies. HBcG vectors may contribute to the further development of versatile HBc VLP-based vaccine and gene therapy applications.

## Introduction

Hepatitis B virus (HBV) core (HBc) virus-like particles (VLPs) are one of the oldest [1–3] and the most powerful protein engineering tools utilised to (i) expose immunological epitopes and/or cell-targeting signals and (ii) package poly- and oligonucleotides as genes and immune stimulatory sequences (for review see [3–8]). HBc VLPs and their numerous derivatives are efficiently produced in bacterial and yeast expression systems (for references see [5–8]). Novel advanced purification and packaging protocols permit the highly technological production and efficient quality control of recombinant HBc-derived VLPs [9].

Bacteria-produced HBc VLPs are represented by two isomorphs with triangulation numbers *T* = 4 and 3 that consist of 240 and 180 HBc monomers and are 35 and 32 nm in diameter, respectively [10]. The three-dimensional structure of the *T* = 4 particles was resolved by X-ray crystallography [11], whereas a quasi-atomic pattern of the *T* = 3 isomorph was reconstructed by docking the dimers of the *T* = 4 crystal structure [12]. Analogous HBc VLP structures have been produced in other efficient heterologous expression systems, including yeast *S. cerevisiae* [13, 14] and *P. pastoris* [15, 16].

The HBc protein consists of two linearly separated domains: (i) the N-terminal self-assembly (SA) domain at amino acid (aa) residues 1–140, which is necessary and sufficient for the protein to self-assemble and result in the structure revealed by X-ray [11], and (ii) the protamine-like arginine-rich C-terminal (CT) domain at aa 150–183 [17], whose three-dimensional structure is unresolved. The SA and CT domains are separated by a hinge peptide 141–149 [18, 19].

The SA domain involves the so-called major immunodominant region (MIR), the most protruding aa residues 78–82 of which are located on the tips of the HBc spikes [11]. The MIR is generally used for the insertion of foreign B cell epitopes to maximally expose these epitopes on the VLP surface and consequently provide the most efficient immunogenic activity (for review see [4–6]).

During HBV life cycle, the CT domain is primarily responsible for the encapsidation of the 3.5-kilobase pregenomic HBV mRNA, which is converted further into partially double-stranded HBV DNA (for a recent review see [20]) and is dispensable for self-assembly [21]. Therefore, so-called HBc∆ particles fully deprived of the CT domain or carrying shortened CT domain fragments are highly efficiently synthesised in bacteria and are consequently often used as the preferred HBc carriers [22].

The nucleic acid-binding sites in the CT domain are organised into four arginine blocks [23] that are buried within HBc VLPs [24]. Although some data demonstrate that CT domain elements may appear on the HBc VLP surface [25–27], the C-terminal insertions of foreign epitopes, in contrast to the MIR and N-terminal insertions, demonstrate generally low immunogenicity in experimental animals (for more detail see [4, 5, 28]). However, the extremely high capacity of the C-terminal insertions [29] has inspired further attempts to elucidate their potential applicability.

In this study, we constructed a novel class of HBc VLP carriers, so-called HBcG vectors, in which arginine residues of the CT domain are fully or partially replaced by glycine residues. The elimination of positively charged CT stretches in the HBcG carriers prevents the encapsidation of bacterial RNA by cultivation in *Escherichia coli* and allows the exposure of a C-terminally inserted model epitope, namely, the major epitope of the HBV preS1 sequence, onto the outer surface of HBcG-derived VLPs. This exposure markedly improves the immunogenicity of the inserted epitope in experimental animals.

## Materials and Methods

### Bacterial Strains

Two *E. coli* strains—*E. coli* K802 (F^−^ r_K_^−^ m_K_^+^*e14 McrA metB1lac Y1 [or lacI*-*Y6] galK2 galT22 glnV44 mcrB*) and BL21 [F^−^*ompT hsdS*B (rB^−^ mB^−^) *gal dcm lon*] were used to produce VLPs.

### Construction of Plasmids for the Expression of HBc- and HBcG-Derived Constructions

**Fig. 1:**
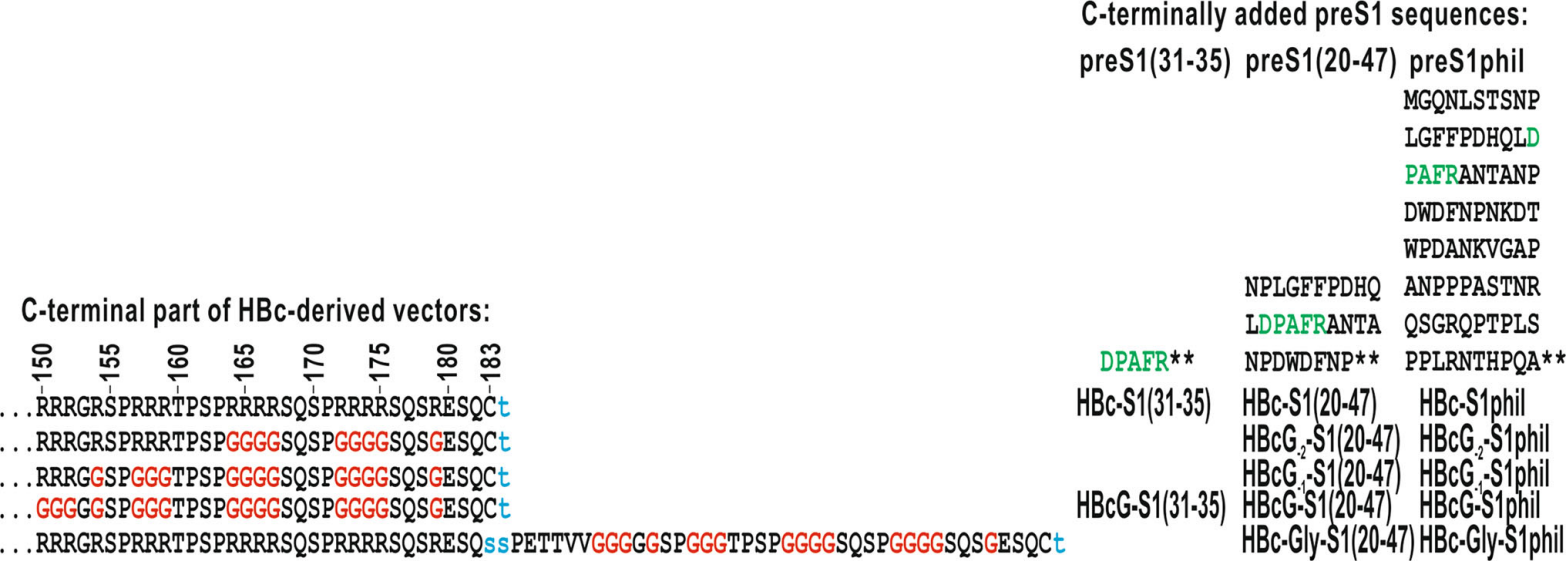
Schematic representation of the HBc- and HBcG-based derivatives carrying three forms of the preS1 epitope. The glycine substitutions are shown in *red*, the aa linkers that are not present in the original sequences are indicated in *lowercase blue*

Two original plasmids were used to construct “one-tail” HBc variants: pHBc183, which expresses the full-length HBc gene [22], and pHBcG, in which all HBc arginine codons starting at position 150 were replaced with glycine codons (primers not shown). For C-terminal insertions into both HBc vectors, a *Bsp1407*I site was introduced after the 183 aa position within the HBc gene using a site-directed mutagenesis kit (Agilent Technologies, Santa Clara, CA, USA) and primers *Bsp1407*Ifor and *Bsp1407*Irev (Table 1). The obtained plasmids were named pHBc183T and pHBcGT for variants with HBc or HBcG genes, respectively. The preS1(30–36) fragment was obtained via the annealing of two primers, DPAFR-*Bsp1407*Ifor and DPAFR-*Bsp1407*Irev, which were then cloned into the pHBc183T/*Bsp1407*I and pHBcGT/*Bsp1407*I vectors to yield plasmids pHBc-S1(31–35) and pHBcG-S1(31–35), respectively (Fig. 1). The preS1(20–47) and preS1phil (containing preS1 regions 12–60 and 89–119) coding fragments were obtained by PCR using primers 5, 6 and 7, 8 (Table 1) and plasmid pHBcΔ-pre-S1(20–47) for fragment preS1(20–47) and plasmid pHBcΔ-pre-S1phil for fragment pre-S1phil, respectively [30, 31]. The PCR fragments were treated with *Bsp1407*I and cloned into the *Bsp1407*I-treated vectors pHBc183T and HBcGT, resulting in plasmids pHBc-S1(20–47), pHBc-S1phil, pHBcG-S1(20–47), and pHBcG-S1phil (Fig. 1). To generate constructs based on HBc in which the first or first two arginine blocks are retained at the HBc C-terminus, site-directed mutagenesis was performed using plasmid pHBcG-S1(20–47) and pHBcG-S1phil and primers 9 and 10 (Table 1) to obtain plasmids pHBcG_−1_-S1(20–47) and pHBcG_−1_-S1phil. Site-directed mutagenesis with the latter plasmids and primers 11 and 12 (Table 1) resulted in plasmids pHBcG_−2_-S1(20–47) and pHBcG_−2_-S1phil, respectively. The pHBc183 plasmid was used for “two-tail” constructs based on HBc with a C-terminal extension—the last cysteine codon in this plasmid was substituted by a serine codon, and an additional serine codon was added followed by a coding sequence for aa 144–183 of HBc in which arginine codons were substituted by glycine codons. An *Ecl136*II site was introduced to clone this second glycine-containing “tail,” which resulted in the pHBc*Ecl136*II plasmid. Treating pHBc*Ecl136*II and pHBcG-S1(20–47) with *Bsp1407*I/*Sac*I yielded the pHBc-Gly-S1(20–47) plasmid, and treating pHBc*Ecl136*II and pHBcG-S1phil with Bsp1407I/*Bsp68*I yielded the pHBc-Gly-S1phil plasmid. All enzymes were from Thermo Fisher Scientific (Waltham, MA, USA).

**Table 1:** List of primers used for plasmid construction

1	*Bsp1407*Ifor	5′GAATCTCAATGTACATAGTAAGGATCCT
2	*Bsp1407*Irev	5′AGGATCCTTACTATGTACATTGAGATTC
3	*DPAFR*-*Bsp1407*for	5′GTACAGATCCAGCCTTCAGGTAGTAAT
4	*DPAFR*-*Bsp1407*rev	5′GTACATTACTACCTGAAGGCTGGATCT
5	*Bsp1407*I 20-47for	5′CTCAATGTACAAATCCTCTGGGATTCTTTCCCGACCACCAGTTG
6	*Bsp1407*I 20-47rev	5′CTATGTACATTACTAGGGATTGAAGTCCCAATCTGGATTTGC
7	*Bsp1407*I preS1for	5′CTCAATGTACAATGGGGCAGAATCTTTCCA
8	*Bsp1407*I preS1rev	5′CTATGTACATTACTAAGCCTGAGGATGAGTGTTTCTCA
9	SPRR1for	5′GACTACTGTTGTTCGTCGTCGTGGCCGTTCCCCT
10	SPRR1rev	5′AGGGGAACGGCCACGACGACGAACAACAGTAGTC
11	SPRR2for	5′GGCCGTTCCCCTCGTCGTCGTACTCCCTCGCC
12	SPRR2rev	5′GGCGAGGGAGTACGACGACGAGGGGAACGGCC
13	*Ecl136*IIfor	5′CTCGTGAATCTCAGAGCTCAGGAATCTGTACATAGTAAG
14	*Ecl136*IIrev	5′CTTACTATGTACAGATTCCTGAGCTCTGAGATTCACGAG

### Expression of Recombinant Genes and Purification of VLPs

The cells were cultivated in Trp-deficient M9 salt medium (M9-cas) supplemented with 1 % casamino acids, and 0.2 % glucose (BD, Franklin Lakes, NJ, USA), Ap, and Km (at 50 and 10 μg/mL, respectively) without an additional induction of Ptrp or in 2×TY medium containing 20 μg/mL ampicillin and supplemented with 2 g glucose, 3.47 g KH_2_PO_4_, and 18.8 g K_2_HPO_4_ per litre (2×TY+P). The aeration of the medium was ensured by incubating 750 mL Erlenmeyer flasks filled with 300 mL of medium on an orbital shaker at 200 rpm. The cultures were incubated for 14–16 h at 37 °C and allowed to reach a final OD540 of 5–8 or 8–10 (in M9-cas media or 2×TY+P media, respectively).

To assess HBc protein synthesis, 2 OD units of cells were pelleted and lysed in 200 μL of Laemmli buffer for 10 min at 100 °C, and 10 μL of the lysate was subjected to SDS-PAGE.

The VLPs were purified from wet fresh cells according to basic methods published elsewhere [10] with some modifications specific to the groups of VLPs. The first group of chimeric VLPs based on wt HBc (named as HBc-derived proteins) and the HBcs containing arginine substitutions by glycine in all arginine blocks (named as HBcG-derived proteins) were purified as follows: 2 g of wet fresh cells was incubated for 30 min on ice in 8 mL of lysis buffer containing 50 mM Tris–HCl, pH 8.0, 5 mM EDTA, 50 µg/mL PMSF, 0.5 M urea, and 0.1 % Triton X-100. The suspension was ultrasonicated five times at 22 kHz at 15-s intervals on ice. After clarification at 7000 rpm for 10 min, the supernatant was incubated at 50 °C for 30 min, repeatedly centrifuged at 7000 rpm for 15 min and loaded onto a Sepharose CL-4B column or Sephacryl S-1000 column (2.5 × 85 cm). PBS buffer without Triton X-100 was used for elution. The HBc-containing fractions (detected by SDS-PAGE) were pooled, and the proteins were precipitated by centrifugation at 52,000 rpm on Beckman 70Ti rotor for 60 min at 4 °C. The resultant pellet was dissolved in 40 mM phosphate buffer supplemented with NaCl to a final concentration of 150 mM. Proteins with final concentrations of 10–20 mg/mL in 50 % glycerol were stored at −20 °C until use. A second group of chimeric VLPs based on HBc containing one arginine block (first of four arginine blocks, named HBcG_−1_-derived proteins) and two arginine blocks (first two of four arginine blocks, named HBcG_−2_-derived proteins) at the C-end were purified as follows: 8 g of wet fresh cells was resuspended in 4 volumes of the lysis buffer (50 mM Tris–HCl, pH 8.0, 5 mM EDTA, 150 mM NaCl, 50 µM PMSF, and 0.1 % Triton X-100) and ultrasonicated 8 times at 22 kHz for 10 s. After clarification of the lysate at 10,000×*g* for 30 min, the soluble proteins were precipitated with 10 % ammonium sulphate at 4 °C for 1 h, followed by centrifugation at 10,000×*g* for 30 min. VLPs in the supernatant were precipitated with 35 % ammonium sulphate at 4 °C overnight, followed by centrifugation at 10,000×*g* for 30 min. The sediment was dissolved in 15 mL of PBS buffer containing 0.5 M urea and 50 µM PMSF and subjected to size-exclusion chromatography on a Sepharose 4 Fast Flow (GE Healthcare, Sweden) 320 mL column (25 × 850 mm) at a flow rate of 0.5 mL/min.

The semi-preparative purification the HBcG-S1phil for the detailed immunological characterisation was performed as follows: 9 g of wet fresh K802 cells was incubated for 30 min on ice in 36 mL of lysis buffer containing 50 mM Tris–HCl, pH 8.0, 5 mM EDTA, 50 µg/mL PMSF, and 0.1 % Triton X-100. The suspension was ultrasonicated five times at 22 kHz at 15-s intervals on ice. After centrifugation at 12,000 rpm for 60 min, 40 mL of the lysate was obtained and supplemented with NaCl to a final concentration of 0.5 M and 40 % PEG8000 water solution at a ratio 3:1, i.e. 13.3 mL. After an overnight incubation, the HBcG-S1phil VLPs were extracted from the pellet using small consecutive aliquots of NET buffer (20 mM Tris–HCl, pH 7.8, 5 mM EDTA, and 0.15 M NaCl). Sixteen millilitres of the extract was loaded onto a Sepharose CL-4B column (2.5 × 85 cm) and eluted with the NET buffer. The VLP-containing fractions (58 mL in total) collected after the PAGE analysis were supplemented with ammonium sulphate to 60 % saturation. The mixture was incubated at 4 °C overnight. The pellet obtained after centrifugation at 10,000 rpm for 30 min was resuspended in 3 mL of 0.1 M Na_2_CO_3_ and 2 mM DTT, loaded onto a Sephacryl S300 (GE Healthcare Europe GmbH Suomen sivuliike, Helsinki, Finland) column (1 × 50 cm), and eluted using the same solution of 0.1 M Na_2_CO_3_ and 2 mM DTT at a velocity of 1.2 mL/h (60 min/1.2 mL fraction). The VLP-containing fractions were identified using OD measurements and PAGE and pooled. A carbonate-neutralising buffer (250 mM HEPES, 500 mM NaCl, 10 mM DTT, and 300 mM MgCl_2_ at pH 4.6) was added in an amount 1/4th of the fraction volume. For storage, half the material was dialysed against the NET buffer with glycerol at a volume/volume ratio of 1:1, and the remaining half was precipitated by adding ammonium sulphate to 50 % saturation for 20 h at 4 °C (for more details see [9]). One day before the first immunisation or following boosts, 1-mL portions of the HBcG-S1phil VLPs were gel filtered on an analytical Sepharose CL-4B column (0.8 × 32 cm). The VLP quality in the pooled fractions was controlled by electron microscopy prior to the immunisation of mice.

The “two-tail” VLPs, i.e. HBc-Gly-S1(20-47) and HBc-Gly-S1phil, were purified as described for the second group of proteins (see above) or as described for HBcG-S1phil, omitting the precipitation by ammonium sulphate of the gel filtration fractions. Although both methods produced particles of similar size according to dynamic light scattering (DLS), VLPs obtained using the second method were less aggregated according to the electron microscopy (EM) tests; these particles were also more reactive in a competitive ELISA than particles obtained by the first method.

### Detection of Protein and Nucleic Acids

The protein concentration was generally determined using the Bradford method. The amount of HBc-Gly-S1(20–47) and HBc-Gly-S1phil proteins used for the competitive ELISA was estimated according to [32]. The Vector NTI 10.0.1 (Thermo Fisher Scientific, Waltham, MA, USA) software package was used to calculate that one optical absorbance unit of empty VLPs corresponds to 0.71 mg of HBc protein.

For native agarose gel electrophoresis (NAGE), the samples were resolved in 1 % UltraPure agarose (Thermo Fisher Scientific, Waltham, MA, USA) in TBE buffer, and the gels were subsequently stained. Specifically, the gels were first stained with ethidium bromide (5 µL of a 10 mg/mL stock in 100 mL of PBS) and then with Coomassie Blue R-250 (60 μg/mL of Coomassie Blue R-250 in 10 % acetic acid). All chemicals were obtained from Sigma–Aldrich (St. Louis, MO, USA).

The protein samples were analysed on 15 % SDS-PAGE gels in a Tris–glycine-SDS system with subsequent Coomassie or silver staining according to standard procedures (LKB Laboratory Manual).

### Electron Microscopy and Dynamic Light Scattering Analysis

For electron microscopy, VLPs in PBS were adsorbed to carbon-formvar-coated copper grids and negatively stained with a 1 % aqueous solution of uranyl acetate. The grids were examined using a JEM-1230 electron microscope at 100 kV or a JEM-100C electron microscope at 80 kV (both Jeol Ltd., Tokyo, Japan).

The DLS analysis was performed on a Zetasizer Nano ZS instrument (Malvern Instruments Ltd, Malvern, UK). The results were analysed using the DTS software (Malvern, version 6.32).

### Antigenicity Test

For the competitive ELISA, 96-well microplates were coated with preS1 peptide p20-47 using 100 μL of peptide solution (10 μg/mL) per well. After incubation overnight at 4 °C, the plates were blocked with 1 % BSA in PBS for 30 min at 37 °C. Subsequently, 50-μL aliquots of serial dilutions of competing protein and 50 μL of the monoclonal antibody MA18/7 [33] were simultaneously added to the wells; to this end, the MA18/7 dilution was used, which resulted in an OD492 value within the range of 0.5–0.6 for the control sample (without competing protein). After incubation for 1 h at 37 °C, the plates were washed three times with PBS containing 0.05 % of Tween-20. Thereafter, 100 μL of horseradish peroxidase-conjugated anti-mouse antibody (Sigma–Aldrich St. Louis, MO, USA) was added at a 1:10,000 dilution. After incubation for 1 h at 37 °C, the plates were washed as before, and OPD substrate (Sigma–Aldrich St. Louis, MO, USA) was added to develop the colour. The per cent inhibition (*I* %) of antibody binding by the competing protein was calculated as follows:

$$I\% = \, \left[ {\left( {{\text{OD}}492\;{\text{test}}\;{\text{sample}}\,{-}\,{\text{OD}}492\;{\text{negative}}\;{\text{control}}} \right)/\left( {{\text{OD}}492\;{\text{positive}}\;{\text{control}}\,{-}\,OD492\;{\text{negative}}\;{\text{control}}} \right)} \right]\, \times \,100.$$

The molar amounts of the proteins necessary for 50 % inhibition (*I*_50_) were calculated.

### Immunisation of Mice

Female BALB/c mice aged 6–8 weeks were obtained from the Latvian Experimental Animal Laboratory (LEAL) at the Riga Stradins University (Latvia). The mice were allocated to groups (five mice per group) and subjected to immunisation by all proteins. Mice from Taconic Biosciences (Hudson, NY, USA) were used for immunisations with HBcG-S1phil formulated in different adjuvants. The animals were maintained at the Latvian Biomedical Research and Study Centre under pathogen-free conditions. The experiments were approved by the Latvian Animal Protection Ethics Committee and the Latvian Food and Veterinary service (Permission No. 31/23.10.2010). Mice obtained from LEAL were subcutaneously immunised on days 0, 14, and 28 with 25 μg of protein in PBS formulated with 250 μg Alhydrogel in a total volume of 0.2 mL per mouse. Mice obtained from TAC were subcutaneously (s.c.) or intraperitoneally (i.p.) immunised with 25 µg of HBcG-S1phil (in PBS) formulated with adjuvants in a total volume of 0.2 mL, using PBS to normalise the volume. The following adjuvants were used in this experiment: 250 μg of Ahydrogel for group A (Brenntag Biosector, Frederikssund, Denmark), 250 μg of Silica for group B (Sigma–Aldrich St. Louis, MO, USA), Complete Freund’s adjuvant (Sigma–Aldrich St. Louis, MO, USA) for the first injection and Incomplete Freund’s adjuvant for the following two injections for group C, and Incomplete Freund’s adjuvant for all three injections for group D. To immunise mice in groups C and D, the protein was mixed with adjuvant at a *v*/*v* ratio of 1:1. Group E of mice was immunised with protein in PBS without any adjuvant. The mice were injected on days 0, 14, and 28, and sera were collected on day 42 from mice sacrificed by cervical dislocation. The anti-HBc and anti-preS1 titres in the sera were determined with a direct ELISA using plates coated with the full-length HBc protein or preS1 peptide p20-47. The end-point titres were defined as the highest serum dilution that resulted in an absorbance value three times greater than that of the control sera obtained from unimmunised mice.

### Statistical Analysis

Statistical analysis was performed by t test that compares the means of two groups (GraphPad Software, http://www.graphpad.com/quickcalcs/ttest1/). Significance levels are indicated by *p*; *p* values <0.05 were considered significant.

## Results

### Construction of a Set of the HBcG Vectors and Preparation of HBc- and HBcG-Derived VLPs

Figure 1 presents the structure of the HBcG vectors based on the HBc protein from the HBV320 genome (genotype D1, subtype ayw2, GenBank accession number X0249675) [34], which included the added preS1 sequences, as well as the parallel paired preS1-carrying constructs based on the natural HBc. The HBcG vectors can be divided into four categories: (i) all four C-terminal arginine stretches are replaced by glycine residues, (ii) the first arginine stretch is preserved, (iii) the two first arginine stretches are preserved, or (iv) the natural C-terminus is prolonged by the C-terminal sequence with fully replaced arginine stretches.

The HBcG vectors and the paired HBc-based constructs are provided with the major virus-neutralising preS1 epitope 31-DPAFR-35 [35, 36] that is recognised by the monoclonal MA18/7 antibody [33]. The preS1 epitope is used in three different forms: (i) the pure epitope, which corresponds to aa residues 31–35, (ii) the 20–47 aa sequence that extends the epitope from both sides, and (iii) the preS1phil structure that contains preS1 fragments 12–60 and 89–119 aa, which corresponds to the full-length hydrophilic component of the preS1 sequence from the HBV320 genome but lacks the hydrophobic preS1 stretch at aa 61–88 [30].

**Fig. 2:**
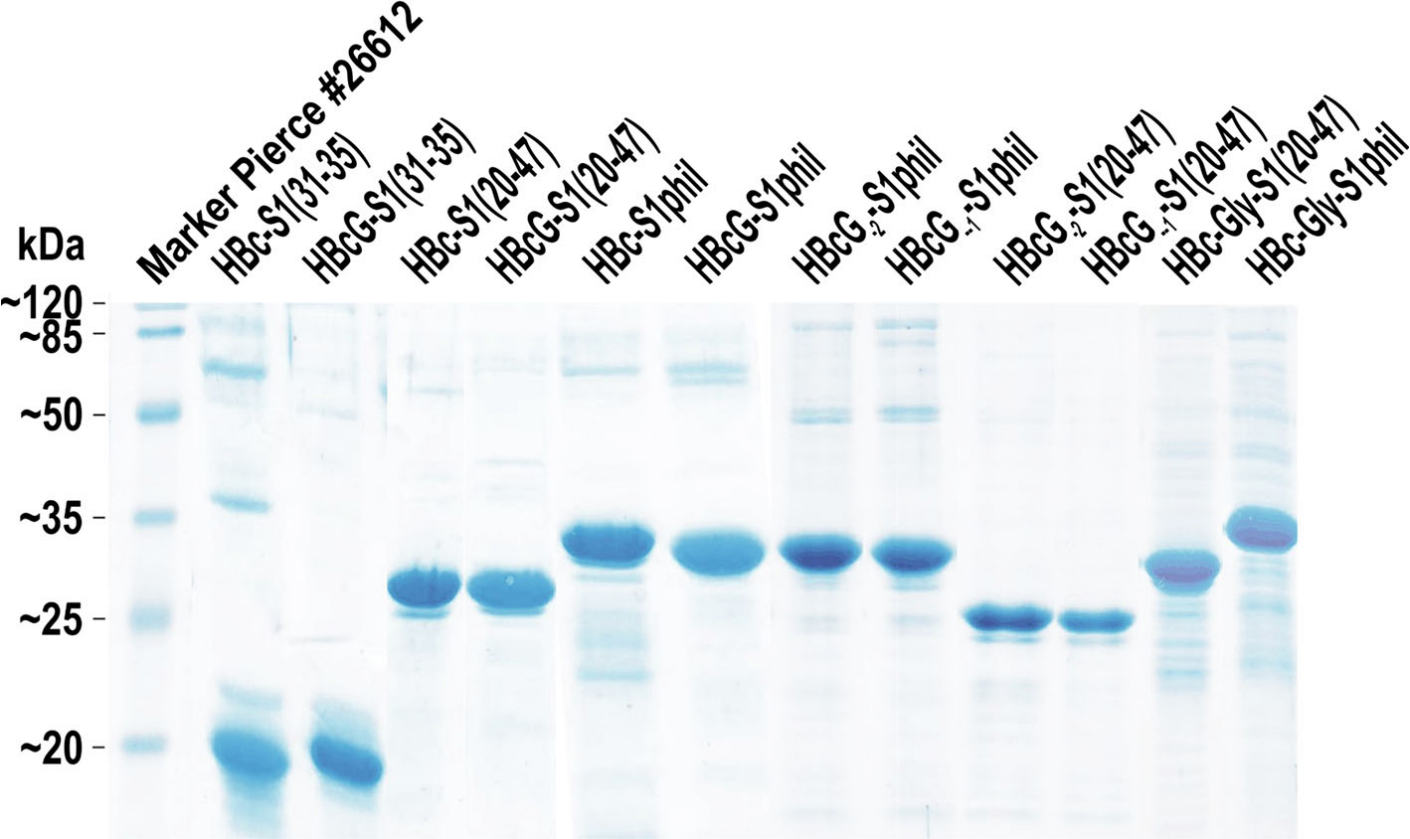
SDS-PAGE analysis of the purified HBc- and HBcG-derived VLPs

**Fig. 3:**
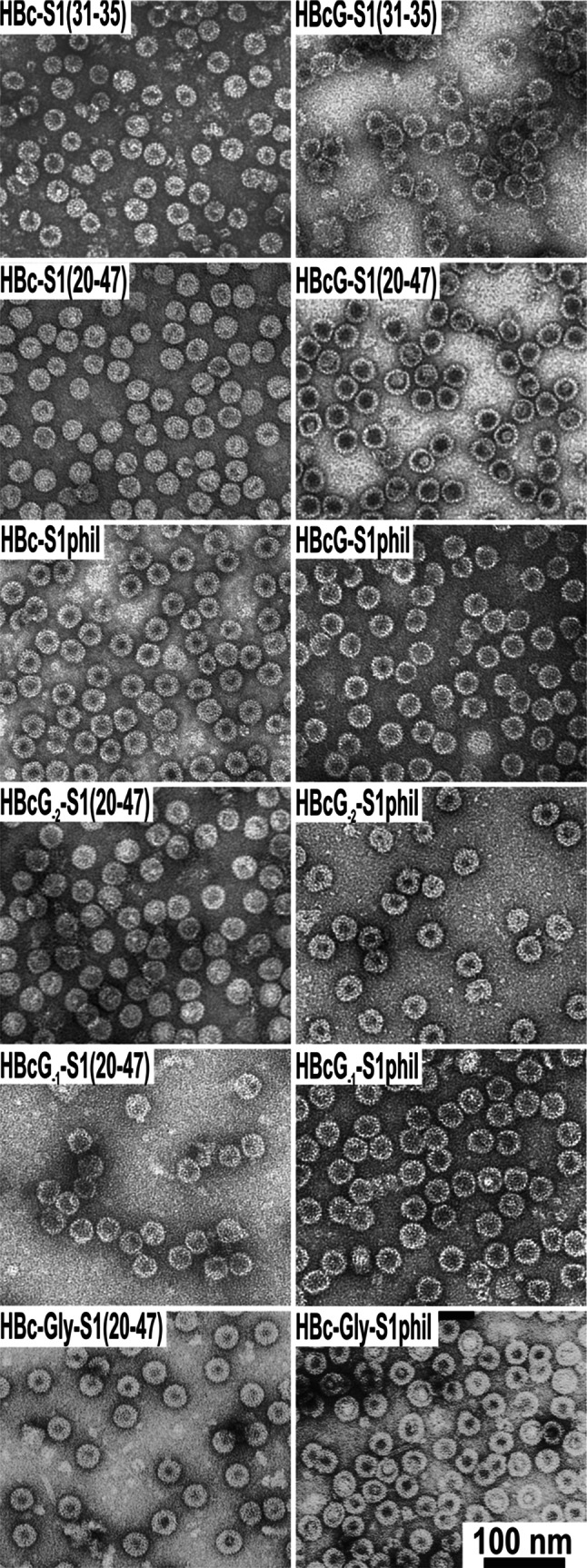
Electron microscopy analysis of the purified HBc- and HBcG-derived VLPs

The HBcG-preS1 VLPs and the analogous HBc-preS1 VLP paired constructs were produced in *E. coli* and purified using a combination of traditional column chromatography methods. The VLP preparations demonstrated high consistency based on native agarose gel electrophoresis (NAGE) (not shown) and SDS-PAGE analysis (Fig. 2). In all cases, the DLS measurements revealed VLP diameters of approximately 35 nm, in accordance with the expected size (not shown). The EM analysis revealed the high quality of HBcG-derived VLPs. (Fig. 3). The VLPs formed by HBc-Gly-S1(20–47), especially the HBc-Gly-S1phil constructs that contained two C-terminal “tails,” demonstrated a definite predisposition to aggregation.

As expected, the NAGE and UV spectra of the purified VLP preparations indicated the presence of the packaged bacterial RNA for HBc-derived proteins, which contain a natural arginine-rich CT domain, and this bacterial RNA was absent when arginine residues were replaced by glycine residues (not shown).

### Location of the C-terminally Added preS1 Epitope on the HBc- and HBcG-Derived VLPs

**Fig. 4:**
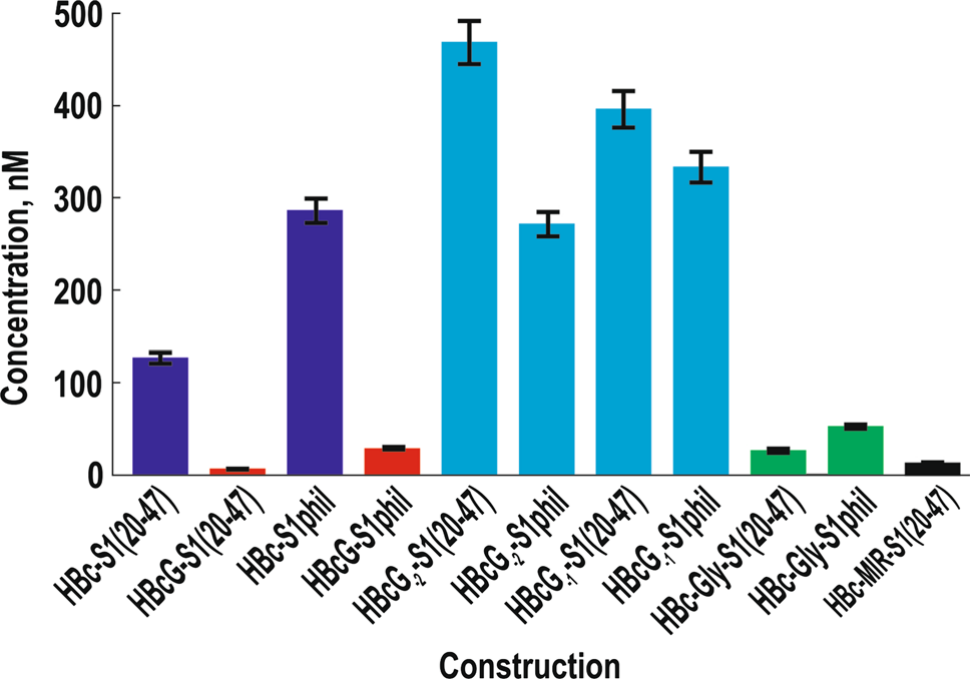
The preS1 antigenicity of the VLPs formed on the basis of the HBc and HBcG vectors. The VLP concentrations necessary and sufficient to inhibit 50 % of the binding of MA18/7 to the 20–47 peptide on the support during the competitive ELISA. Tests were done in triplicate. Significance of differences between all of groups was less than 0.035. The *bars* for VLPs based on HBc-, HBcG-, and two-tail HBc-Gly-derived vectors are shown *blue*, *red*, and *green*, respectively. The *bars* for VLPs based on partially replaced arginine stretches are shown in *light blue*. The *bar* for the HBc-MIR-derived construction that served as a control is shown *black*

The antigenicity of the HBcG-preS1 VLPs and accessibility of the inserted preS1 epitope to the specific antibody was characterised using a competitive ELISA with the monoclonal MA18/7 antibody and compared to the antigenicity of the appropriately paired HBc-preS1 constructs (Fig. 4). The chimeric HBc VLPs carrying the preS1(20–47) epitope insertion at the MIR were used as control. The HBcG-S1(20–47) demonstrated a competition level that was very similar to that of the chimeric HBc VLPs carrying preS1 insertions within the MIR. The HBcG-S1phil competed to a slightly lesser extent, but this competition was, nevertheless, more efficient than that of the paired construct, HBc-S1phil. The competition lag of HBcG-S1phil compared to HBcG-S1(20–47) may be due to the remarkable length of the added preS1 sequence (80 aa); where specifically, the preS1 epitope 31–35 is located at the N-terminal part of the addition and can consequently be shielded by the C-terminal part of the addition. The HBcG-preS1 VLPs carrying the shortest version of the preS1 epitope, namely, aa residues 31–35, do not demonstrate any competition for MA18/7 antibodies, and this behaviour is similar to that of the HBc VLPs that lack preS1 insertions, which served as a negative control (not shown).

### Immunogenicity of the HBcG-Derived VLPs in Mice

**Fig. 5:**
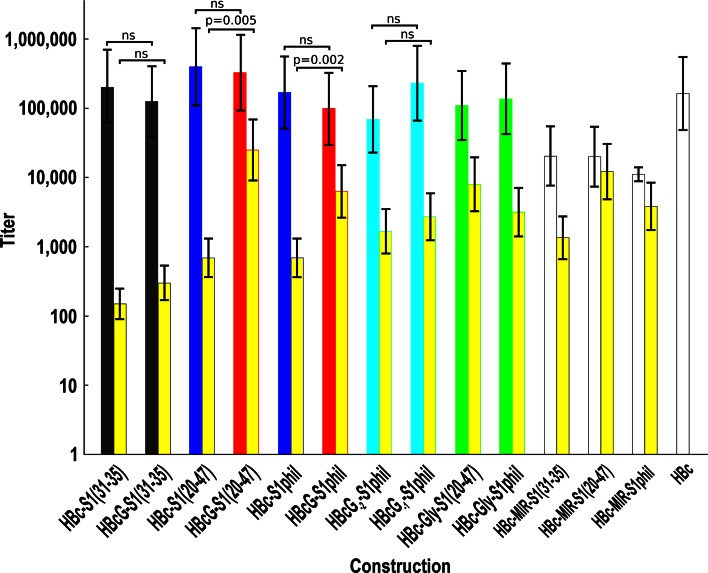
The immunogenicity of a set of the HBcG-derived VLPs compared with that of the appropriate HBc-derived VLP pairs and HBc VLPs carrying preS1 insertions within the MIR. Mice obtained from LEAL were immunised with Alhydrogel adjuvant as stated in the “Materials and Methods” section. The *colour* of the anti-HBc *bars* corresponds to that used in Fig. 4 for the respective constructs. The anti-HBc *bars* for the constructs carrying preS1(31–35) sequences are *grey*. The anti-HBc *bars* corresponding to HBc-MIR-derived constructs and to the HBc without any insertions are *white*. The anti-preS1 *bars* are *yellow* but are outlined by a *coloured contour* corresponding to the construct in question. The presented results are the mean values (±SD) of five individual mouse sera. Significance levels for paired constructs are indicated by *p*, *ns* nonsignificant

The HBcG-derived VLPs carrying the preS1(20–47) and preS1phil sequences demonstrated much higher anti-preS1 antibody responses in mice than did the paired HBc-derived VLPs (Fig. 5). Specifically, the latter did not induce remarkable anti-preS1 antibody levels. The HBcG-S1(20–47) VLPs induced higher anti-preS1 titres than the HBcG-S1phil; this finding correlated well with the antigenicity data, which demonstrated that the accessibility of the preS1 epitope was improved over the native state. The anti-preS1 antibody titres and the ratio of the preS1(20–47) and preS1phil additions were similar for the constructs in which the C-terminal sequence with fully replaced arginine residues followed the natural HBc C-terminus, i.e. constructs HBc-Gly-S1(20–47) and HBc-Gly-S1phil. The constructs in which the first arginine stretch (HBcG_−1_-S1phil) and two first arginine stretches (HBcG_−2_-S1phil) were preserved elicited lower anti-preS1 responses than the VLPs that contained the fully substituted arginine residues at the C-terminus.

Generally, the anti-preS1 response elicited by HBcG-derived VLPs carrying preS1(20–47) and preS1phil sequences was comparable to the responses induced by analogous constructs carrying the respective preS1 sequences within the MIR (Fig. 5).

The HBcG-S1(31–35) VLPs and the paired HBc-S1(31–35) were able to elicit only very low anti-preS1 antibody responses (Fig. 5).

The anti-HBc response induced by HBcG-derived VLPs did not markedly differ from the anti-HBc response induced by native HBc VLPs (Fig. 5).

**Fig. 6:**
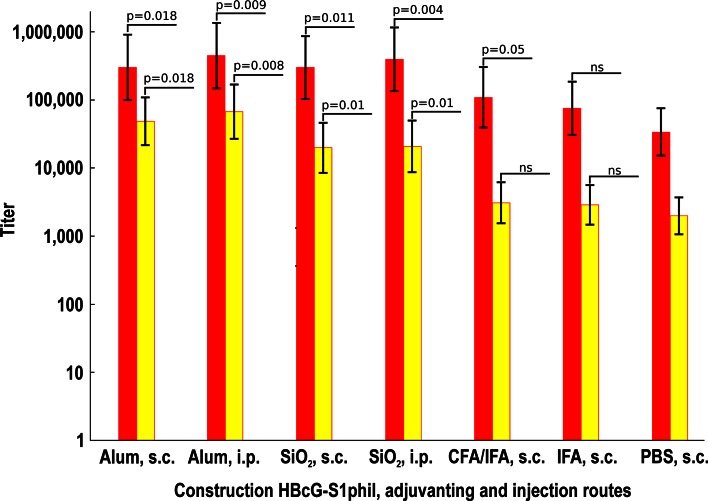
The immunogenicity of the HBcG-S1phil VLPs by different adjuvanting conditions and immunisation routes. Mice obtained from TAC were immunised with different adjuvants as stated in the “Materials and Methods” section. The colour of the anti-HBc *bars* is *red*. The *colour* of the anti-preS1 (20-47) *bars* is *yellow*. The presented results are the mean values (±SD) of five individual mouse sera. Significance of differences between groups with adjuvants and PBS group was assessed at the highest dilution and are indicated by *p*, *ns* nonsignificant

The HBcG-S1phil VLPs were selected for the further detailed immunogenicity testing based on alternate immunisation routes and different adjuvant protocols (Fig. 6). First, the resultant data show that the intraperitoneal immunisation route is preferable to the subcutaneous route. Second, the alum adjuvant was more efficient than the silica nanoparticles introduced by us as an adjuvant recently [37]. The formulation of the HBcG-S1phil VLPs in CFA/IFA, IFA/IFA, or PBS resulted in much lower anti-preS1 and anti-HBc responses (Fig. 6).

The anti-preS1 titres were generally obtained by titrating the murine sera on the plates coated using the synthetic preS1(20–47) peptide. When recombinant full-length preS1 protein purified from *E.* *coli* cells (Petrovskis et al., not published) was used for coating, the anti-preS1 titres did not increase. In fact, the titres were lower for the full-length protein than for the preS1(20–47) peptide. This difference may be due to the masking of the major preS1(31–35) epitope by the C-terminal preS1 sequences.

## Discussion

The safe localisation of an inserted foreign epitope on the VLP surface is the first necessary step for the induction of an efficient humoral anti-epitope response after the immunisation of experimental animals by chimeric VLPs (for detailed review see [3–8, 38]). The next important prerequisite consists in the self-assembly quality and the appropriate stability of the constructed chimeric VLPs carrying the foreign epitope in question. Here, we constructed a class of new HBcG vectors that permit the stable exposure of C-terminally added foreign epitopes on the high-quality VLPs and therefore fulfil both above-mentioned requirements. The HBcG vector in which all four C-terminal arginine stretches were replaced by glycine residues demonstrates not only maximal antigenicity (Fig. 4) and immunogenicity (Fig. 5) for the inserted preS1 epitope but also highly stable chimeric HBcG-preS1 VLPs during long-term storage (Renhofa et al., in preparation). Both the antigenicity and immunogenicity of the HBcG-preS1 VLPs was comparable with that of HBc-derived VLPs carrying the preS1 epitope insertions within the HBc MIR (Fig. 5).

The fact that the substitution of the arginine motifs at the C-terminus with alanine residues may induce a conformational change that allows C-terminal insertions to become more exposed and more immunogenic on the VLP surface has been demonstrated recently for the woodchuck hepatitis core (WHc) VLPs by the David Milich group [39]. In contrast to the MIR insertions, the foreign epitopes added to the C-terminus of the HBcG vector were much more flexible because the inserted epitope sequences remained linear with an unfixed C-terminus. This flexibility is especially important when HBcG vectors are used to insert cell receptor-recognising tools to target the packaged VLP nanocontainers to specific cell types. The HBcG vectors carrying one or two residual arginine stretches can present the preS1 epitope on the VLP surface to a lesser extent, but their ability to bind nucleic acids differs. If the HBcG_−2_-S1phil construct binds some nucleic acids, no bands are visible in ethidium bromide-stained agarose gels for the HBcG_−2_-S1(20–47), HBcG_−1_-S1phil, and HBcG_−1_-S1(20–47) proteins. However, the ability to package nucleic acids is an obligatory prerequisite for the recently described use of VLP vectors as putative nanocontainers for genes and/or immunostimulatory oligonucleotide sequences [9]. As expected the ability to package nucleic acids was not reduced in HBc-Gly vectors that retained the native full-length C-terminus extended by a copy of the glycine-exchanged C-terminus, i.e. the so-called “two-tail” constructs HBc-Gly-S1(20–47) and HBc-Gly-S1phil. Notably, these vectors also demonstrated a clear ability to provide the preS1 sequences with definite immunogenic activity (Fig. 5). Like the original HBc C-terminal insertions [3–8, 29], we expected the high capacity of C-terminal insertions in case of HBcG and HBc-Gly vectors. This hypothesis was supported by the successful insertion of the 80-aa-long preS1phil sequence demonstrated herein, which resulted in well self-assembled and stable VLPs (Fig. 3).

The remarkable advantage of the HBcG-based VLPs consists of their ability to endure the removal of the packaged traces of bacterial RNA (Renhofa, preliminary data) via the recently described alkali treatment procedure [9]. This ability promises not only an easier purification procedure but also presents a clear background for the standardisation and quality control of final biotechnological products. However, we need to remember that the VLP-packaged RNAs play a role of the TLR-7 ligand during immune response in mice [40]. Therefore, the knowledge-based providing of the RNA-free VLPs with the respective activities is one of the most important issues for the future.

The HBcG vectors retained the natural structure of the HBc MIR, which is important for cases that require a high immunological anti-HBc response in parallel with a foreign epitope-induced response. Moreover, the HBcG vectors permit the simultaneous application of two efficient sites: the MIR and C-terminus for the simultaneous exposure of two foreign epitopes or a pair of an epitope and a cell-targeting signal.

We selected the preS1 immunodominant epitope as the model epitope in this work. This immunodominant epitope could take the following form: (i) a pure epitope corresponding to aa residues 31–35, (ii) an extended epitope 20–47, and (iii) a preS1phil sequence 12-60 plus 89–119 aa corresponding to the full-length hydrophilic component of the preS1 sequence of the HBV of genotype D1, subtype ayw2 [34], in which the hydrophobic 61–88 stretch was deleted [30]. This major preS1 epitope is recognised by the highly specific antibody MA18/7 [33] and has been mapped as a linear 31-DPAFR-35 stretch [35, 36]. Antibodies elicited against this epitope in immunised animals are strongly virus-neutralising [41].

Below, the detection of the preS1 epitope by the MA18/7 antibody on the VLP surface is elaborated in detail. The preS1 epitope has been used as a model epitope not only for the fine mapping of the HBc VLP surface but also to map the RNA phage Qβ [42], DNA phage T7 [43], potato virus Y [44], polyomavirus [30, 45], human papillomavirus [46], and retrovirus [47] VLPs.

For HBc VLPs, the preS1 sequences 31-DPAFR-35 [12, 42, 48–50], 27–53 [28], 21–47 [51, 52], 20–47 [31], 1–42 [53], preS1phil [30], and the full-length preS1 [54] were inserted into the HBc MIR and appeared on the outer surface of the produced HBc-preS1 VLPs. The exposure on the HBc VLP surface was also demonstrated for N-terminal additions of the preS1 sequences [28, 55, 56]. In our experiments, the C-terminal additions of long preS1 sequences [57–60] resulted in the following: (i) contradictory preS1 antigenicity that strongly depended on the quality of the chimeric VLPs and (ii) negligible anti-preS1 immunogenicity when the quality of VLPs was satisfactory. The low immunogenicity of the C-terminally added preS1 sequences correlated well with the generally low level of exposure and immunological activity of C-terminal additions of many other epitopes to the HBc VLPs (for review see [4–6, 28] ).

Here, both preS1(20–47) and preS1phil sequences were able to induce a remarkable anti-preS1 response when incorporated into HBcG vectors, whereas the pure preS1(31–35) epitope was neither exposed on the VLP surface nor able to induce anti-preS1 antibodies.

Concerning differences of the anti-preS1 titers obtained in ELISA on two different supports, the antigenic quality of the full-length preS1 protein could be lowered by masking of the 20–47 stretch by other protein parts during coating on the solid support.

Because anti-preS1 epitope antibodies are virus-neutralising [41], the chimeric HBcG-preS1 VLPs may serve as prototypes for the generation of a combined therapeutic and prophylactic anti-HBV vaccine in accordance with the current conception of the latter [61]. Moreover, the safe biotechnological background of the purification and standardisation protocols underlines the possible practical application of the elaborated HBcG vectors for the construction of prophylactic and/or therapeutic vaccines as well as advanced cell-targeting and gene therapy tools.
